# Biofabrication of functional bone tissue: defining tissue-engineered scaffolds from nature

**DOI:** 10.3389/fbioe.2023.1185841

**Published:** 2023-08-08

**Authors:** Aaqil Rifai, D. Kavindi Weerasinghe, Gebreselassie Addisu Tilaye, David Nisbet, Jason M. Hodge, Julie A. Pasco, Lana J. Williams, Rasika M. Samarasinghe, Richard J. Williams

**Affiliations:** ^1^ Institute for Mental and Physical Health and Clinical Translation, School of Medicine, Deakin University, Geelong, VIC, Australia; ^2^ The Graeme Clark Institute, The University of Melbourne, Melbourne, VIC, Australia; ^3^ Department of Biomedical Engineering, Faculty of Engineering and Information Technology, The University of Melbourne, Melbourne, VIC, Australia; ^4^ Melbourne Medical School, Faculty of Medicine, Dentistry and Health Science, The University of Melbourne, Melbourne, VIC, Australia; ^5^ Laboratory of Advanced Biomaterials, John Curtin School of Medical Research, Australian National University, Canberra, ACT, Australia; ^6^ Aikenhead Centre for Medical Discovery, St. Vincent’s Hospital, Melbourne, VIC, Australia; ^7^ Barwon Health, Geelong, VIC, Australia; ^8^ Department of Epidemiology and Preventive Medicine, Monash University, Melbourne, VIC, Australia; ^9^ Department of Medicine-Western Health, The University of Melbourne, St Albans, VIC, Australia

**Keywords:** hydrogel, biofabrication, organoid, 3D bioprinting, growth factor, bone tissue, tissue engineering and regenerative medicine, biomaterials

## Abstract

Damage to bone leads to pain and loss of movement in the musculoskeletal system. Although bone can regenerate, sometimes it is damaged beyond its innate capacity. Research interest is increasingly turning to tissue engineering (TE) processes to provide a clinical solution for bone defects. Despite the increasing biomimicry of tissue-engineered scaffolds, significant gaps remain in creating the complex bone substitutes, which include the biochemical and physical conditions required to recapitulate bone cells’ natural growth, differentiation and maturation. Combining advanced biomaterials with new additive manufacturing technologies allows the development of 3D tissue, capable of forming cell aggregates and organoids based on natural and stimulated cues. Here, we provide an overview of the structure and mechanical properties of natural bone, the role of bone cells, the remodelling process, cytokines and signalling pathways, causes of bone defects and typical treatments and new TE strategies. We highlight processes of selecting biomaterials, cells and growth factors. Finally, we discuss innovative tissue-engineered models that have physiological and anatomical relevance for cancer treatments, injectable stimuli gels, and other therapeutic drug delivery systems. We also review current challenges and prospects of bone TE. Overall, this review serves as guide to understand and develop better tissue-engineered bone designs.

## 1 Introduction

Musculoskeletal conditions such as osteoarthritis, rheumatoid arthritis, osteoporosis, and sarcopenia are becoming increasingly prevalent, leading to pain and decreased mobility (WHO, 2022). According to the World Health Organisation, 1.71 billion people suffer from musculoskeletal conditions worldwide (WHO, 2022). The musculoskeletal system is typically defined as the bone (support), connective tissue (anchor), joints (pivots) and muscle (movement). This complex interplay of tissues enables effective locomotion and is vital for quality of life. Bone is a highly integrated component of this system, providing functions beyond mechanical and structural support. As a reservoir of growth factors and cytokines, bone protects internal organs and structures, stores calcium and phosphate, maintains mineral homeostasis, and houses bone marrow (Clarke, 2008; Florencio-Silva et al., 2015). However, bone can be damaged beyond its innate ability to self-repair due to disease, trauma, volumetric loss, or injury. With bone diseases, the common methods of treatment employ auto and allograft procedures, which may lead to a mismatch with the donor cells, site morbidity and further injuries. More appropriate bone substitutes with anatomically defined structures and functions are needed to repair or replace tissues.

**FIGURE 1:**
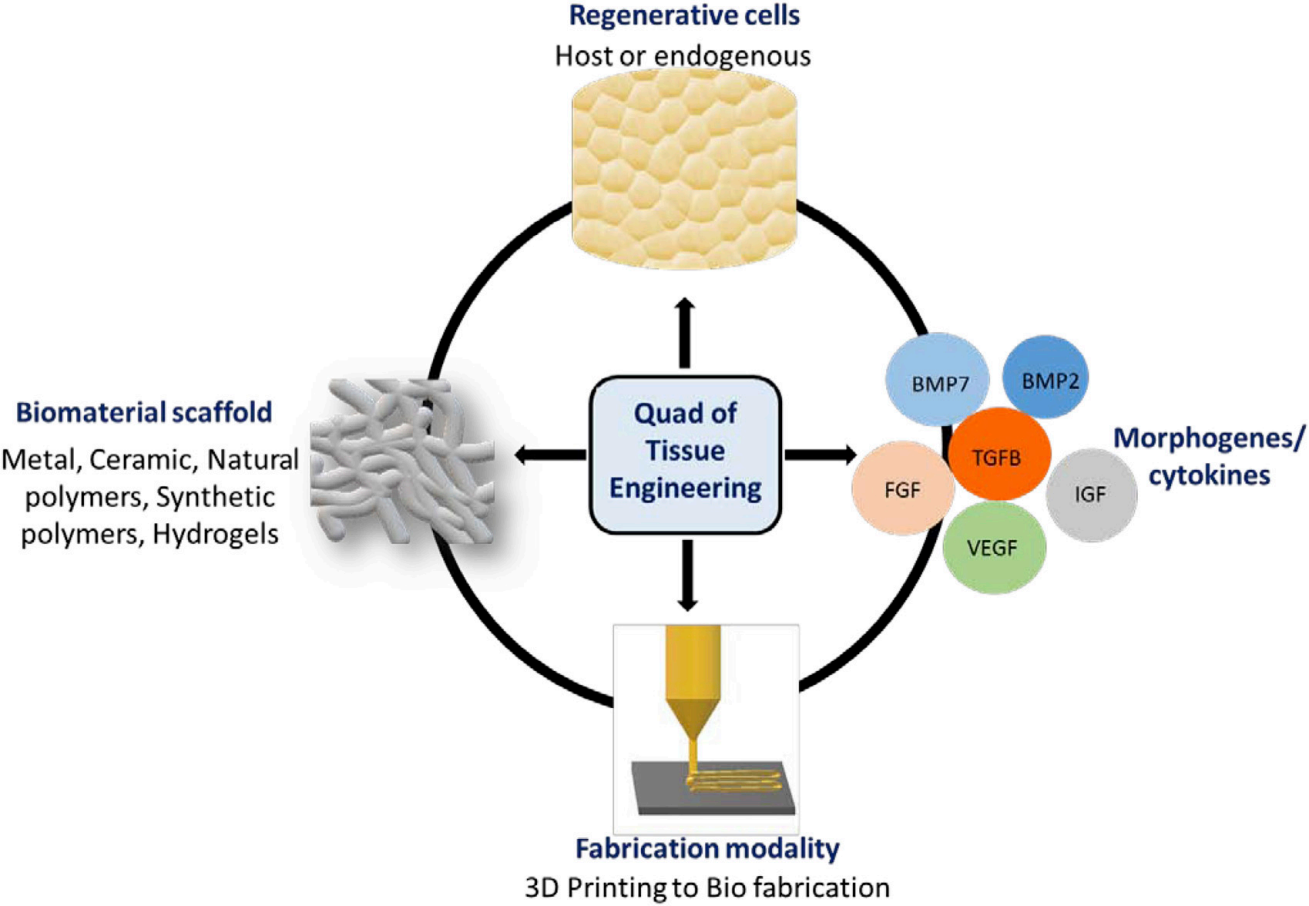
Quad of Tissue Engineering: The biomaterial scaffold, regenerative cells, instructive morphogens/cytokines and fabrication modality.

To repair and regenerate complex organs, including bone, tissue engineering (TE) approaches combine engineering practices, sophisticated materials, and regenerative biology. In this review, we define the “Quad” of TE as: 1) the biomaterial scaffold; 2) fabrication modality; 3) regenerative cell (host or endogenous) and; 4) an instructive morphogen/cytokine (Mironov et al., 2009) (Figure 1). Engineered tissues depend on the quality and function of each member of the Quad, so altering one component can affect the others. Progress in cell and material technologies, such as bioreactors, cell selection tools and 3D printing has led to more efficient therapies for the repair in simple laboratory and pre-clinical models. (Freeman et al., 2020; Collins et al., 2021) However, self-sufficient solutions that enable complete tissue integration and homeostasis remain elusive (Moroni et al., 2018).

Additive manufacturing (colloquially known as 3D printing) has been modified and repeatedly optimised to enable the new technique of biofabrication, whereby a range of methods and materials include engineered living cell/tissue to generate complex and biomimetic constructs. While biofabrication does not completely replicate the structure of native bone tissue, it is versatile, allowing the manufacture of critical mechanical and physical components. Biomaterials are typically inspired by the extracellular matrix (ECM) of bone. Bone ECM is three-dimensional and regulates cell adhesion, proliferation, differentiation, responds to growth factors (GFs), and stimulates new bone formation (Lin et al., 2020). In order to enable the host cells to regenerate, it is necessary to construct a model that mimics the bone’s osteoconductive (providing a strong bone matrix for osteogenesis), osteoinductive (stimulate the differentiation of mesenchymal stem cells towards osteogenic lineage), osteogenic (ability to produce the bone), and vascularisation properties (Albrektsson and Johansson, 2001; Collins et al., 2021). The ECM is defined as the materials that are not part of a cell. The ECM’s main components include glycoproteins (the most abundant collagens), proteoglycans and hyaluronic acid.

Bone tissues rely on morphogens or cytokines, here classified as GFs, at the defect site to allow cells to migrate and initiate the healing process. Functionalisation through these GF molecules in tissue-engineered scaffolds can improve bone regeneration. Table 1 shows the prominent family of molecules involved in bone regeneration. Current strategies focus on osteoinduction, most notably using GFs such as Bone Morphogenetic Protein-2 (BMP2) and −7 (BMP7), which promote differentiation, homeostasis, and self-renewal (Halloran et al., 2020). More recently, bone repair approaches have focused on increasing pathways towards vascularisation using bioactive molecules such as VEGF. However, It is important to strategically deliver molecules according to the defect site. Bone healing is a multi-step process controlled by cytokines, cascading with various cell types, including inflammatory cells, mesenchymal progenitors, vascular cells and osteocytes. Therefore, TE approaches aim to combine cells and biomaterials with GFs for effective tissue repair.

**TABLE 1:** Growth factors/cytokines involved in bone regeneration/osteogenesis.

Growth factor/Cytokine	Biological response	References
IL-1, 6, 8, 15, 17, 23, 34	Osteoclastogenesis	Amarasekara et al. (2018)
IL-3, 4, 10, 12, 27, 33	Anti osteoclastogenesis	Amarasekara et al. (2018)
IL-10, 18	Osteoblastogenesis	Amarasekara et al. (2021)
IL-1, 4, 12, 13, 23	Anti osteoblastogenesis	Amarasekara et al. (2021)
IL-7	Osteoclastogenesis and anti osteoblastogenesis	Amarasekara et al. (2018), Amarasekara et al. (2021)
IL-11	Osteoclastogenesis and osteoblastogenesis	Amarasekara et al. (2018), Amarasekara et al. (2021)
TNFα	Osteoclastogenesis and anti osteoblastogenesis	Amarasekara et al. (2018)
TNFβ	Anti osteoblastogenesis	Amarasekara et al. (2021)
IFNα, IFNβ	Anti osteoclastogenesis and osteoblastogenesis	Amarasekara et al. (2018), Amarasekara et al. (2021)
IFNγ	Anti osteoclastogenesis and osteoblastogenesis	Amarasekara et al. (2018), Amarasekara et al. (2021)
BMP 2,4,6,7	Osteoblastogenesis, chondro-osteogenesis, osteo induction	Devescovi et al. (2008a), Yamaguchi et al. (2008), Lavery et al. (2009), Shen et al. (2010)
TGFβ	Osteoblastogenesis, angiogenesis, immune suppression, ECM synthesis, simulation of cell growth	Devescovi et al. (2008a), Wu et al. (2016)
FGF	Fibroblast proliferation, osteoblastogenesis (FGF2), angiogenesis	Devescovi et al. (2008a), Fei et al. (2011)
Oncostatin M	Osteoblastogenesis	Amarasekara et al. (2021)
VEGF	Osteoblastogenesis, angiogenesis	Devescovi et al. (2008a), Yang et al. (2012)
IGF	Osteoblastogenesis (IGF1), regulation of growth hormones	Devescovi et al. (2008a), Feng et al. (2014)
RANK/RANKL/OPG	Osteoclastogenesis	Boyce and Xing (2008)
Sclerostin	Anti osteoblastogenesis	Lewiecki (2014)

The regenerative cells can be either from the host or introduced from a donor. They respond under appropriate cues such as cell-cell, cell-factor or cell-ECM interactions. These biological processes involve cell condensation, proliferation, differentiation, matrix production, tissue maturation, and the cell’s ability to generate ECM (DuRaine et al., 2015). Cell-cell and cell-ECM interactions can result in a tissue that has relative morphological, and structural similarities, and comparable functional properties to native bone, by recreating fundamental processes that occur *in-vivo* (Athanasiou et al., 2013).

In this review, we will introduce the key features of bone that must be considered to design biological constructs. We provide an overview of the properties of natural bone and the regenerative process involved in fracture healing, maintaining bone structure and the bone remodelling cycle. The therapeutic challenges will be discussed including the pathologies associated with bone defects and the limitations of current treatment options. We then address the importance of TE strategies as an alternative approach with the selection of biomaterials, cells and GFs to generate functional bone tissue. Special attention will be focused on the role of additive manufacturing and biofabrication, as they are believed to play an innovative and important role, facilitating bone repair. Finally, we address the associated challenges and the potential strategies to regenerate the bone.

## 2 Bone biology

### 2.1 The structure function relationship of bone

**FIGURE 2:**
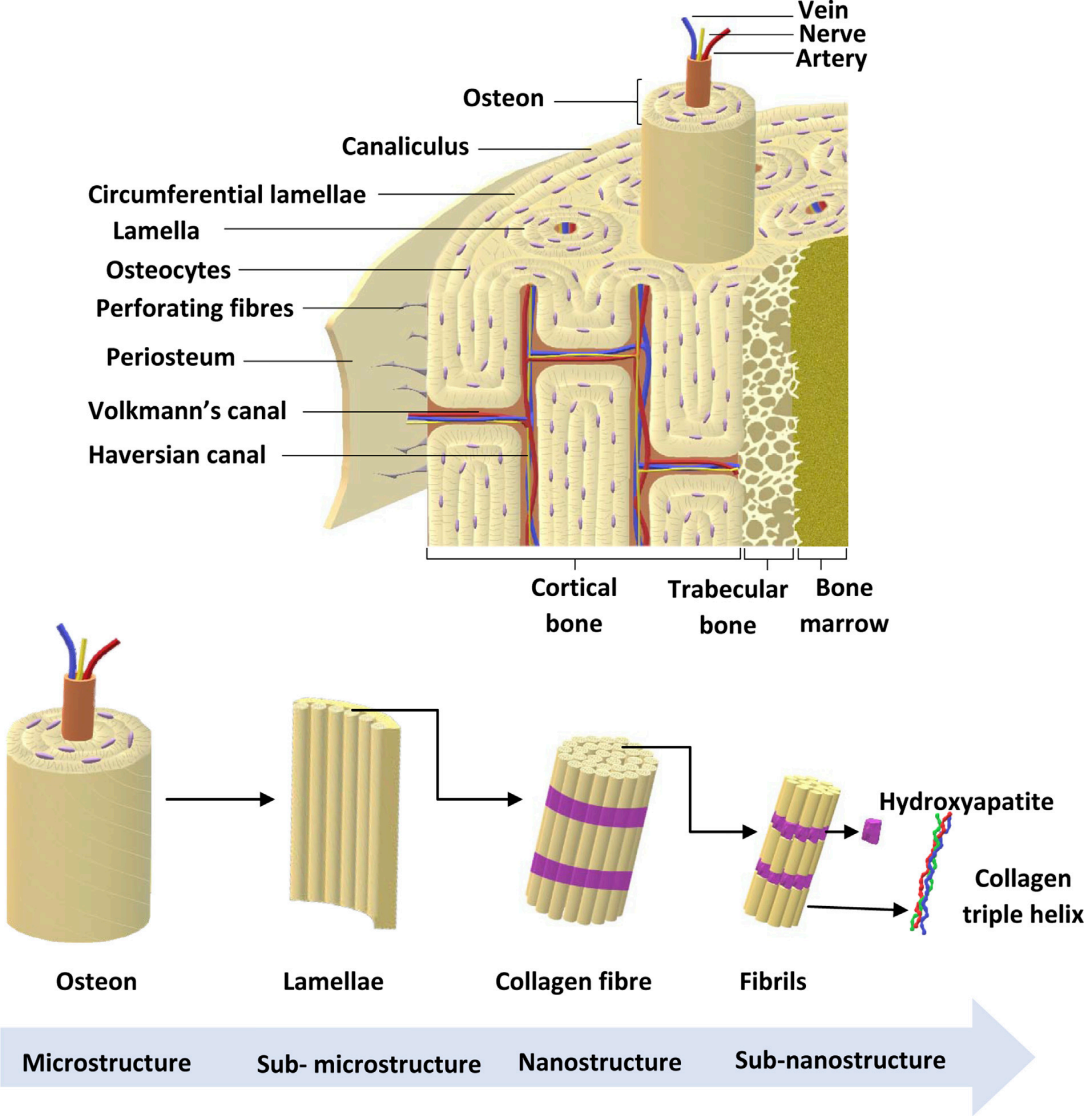
Bone anatomical structure. Bone ECM comprises organic components, inorganic components, cellular components and water. The outer layer of the bone is the periosteum, which consists of greater ECM and fewer cellular components. Cortical bone is found in the diaphysis and is composed of osteons. Trabecular bone is in the metaphysis and epiphysis while endosteum is the inner membrane formed by type III collagen and osteo-progenitor cells. Bone marrow is involved in bone repair and regeneration. Further, bone ECM consists of organic components, inorganic components, cellular components and water (Alvarez and Nakajima, 2009; Fraile-Martínez et al., 2021). Adapted from Henkel et al. (2013).

There are approximately 206 bones in the adult skeleton (Karpiński et al., 2017). Bone is a multicomponent structure consisting of a number of structurally and functionally important subunits, as shown in Figure 2. The bone marrow is a soft, highly vascularised tissue found at the centre of most long bones, and is the primary site for haematopoiesis. Bone marrow produces red blood cells (erythrocytes), some white blood cells, granulocytes and platelets (Karpiński et al., 2017). The ECM consists of 40% organic material (hydrated, flexible) and 60% inorganic material (rigid, strong) (Alvarez and Nakajima, 2009; Lin et al., 2020; Fraile-Martínez et al., 2021). The main component of the organic ECM is collagen type I, which provides mechanical support whilst acting as a scaffold for bone cells. Furthermore, glycoproteins, proteoglycans, and non-collagenous proteins play an important role (Alvarez and Nakajima, 2009; Lin et al., 2020; Fraile-Martínez et al., 2021) that regulate ECM and bone formation by regulating various GFs and cytokines (Bi et al., 2005).

The main component in inorganic ECM is a calcium compound called hydroxyapatite that provides support, stiffness and strength to the bone while also acting as an iron and calcium reservoir (Gopal et al., 2020; Ciosek et al., 2021; Fraile-Martínez et al., 2021). The cellular component of bone contains mesenchymal stem cells, including osteoprogenitors with potential to differentiate into osteoclasts, osteoblasts and osteocytes (Alvarez and Nakajima, 2009; Fraile-Martínez et al., 2021). An individual’s bone structure is composed of diaphysis, a tubular shaft that runs between the proximal and distal ends, metaphysis, the growth plate, and epiphysis, the wider section at each end. Bone is further distinguished as 80% cortical/compact and 20% trabecular/spongy bone (Lin and Kang, 2021). Cortical bone forms the outer layer with 5%–10% porosity, while the trabecular bone forms the inside of the bone with 50%–90% porosity (Lin and Kang, 2021). Due to the differences in porosity and composition, the mechanical properties of cortical and trabecular bone vary; where the cortical bone is more capable of withholding higher stress compared to the trabecular bone when a fracture occurs (Table 2), which is also shown using Youngs modulus (Table 2) (Lin and Kang, 2021). Bone microstructure consists of the haversian canal, osteons and lamellae, while the nanoscale bone consists of fibrillar collagen, and sub-nanoscale bone consists of minerals and collagen (Figure 2) (Qu et al., 2019).

**TABLE 2:** Mechanical properties of cortical and trabecular bone. Adopted from Alvarez and Nakajima (2009).

	Test	Young’s modulus range x 10^9^ N/m^2^	Strength x 10^6^ N/m^2^
Cortical Bone	Compression test	14.1–27.6	219 ± 26 Longitudinal 153 ± 20 Transverse
	Tensile test	7.1–24.5	172 ± 22 Longitudinal 52 ± 8 Transverse
Trabecular Bone	Compression test	0.1–0.4	1.5–9.3
	Tensile test	14.8 ± 1.4	1.6–2.42

### 2.2 The (multi)cellular nature of bone

**FIGURE 3:**
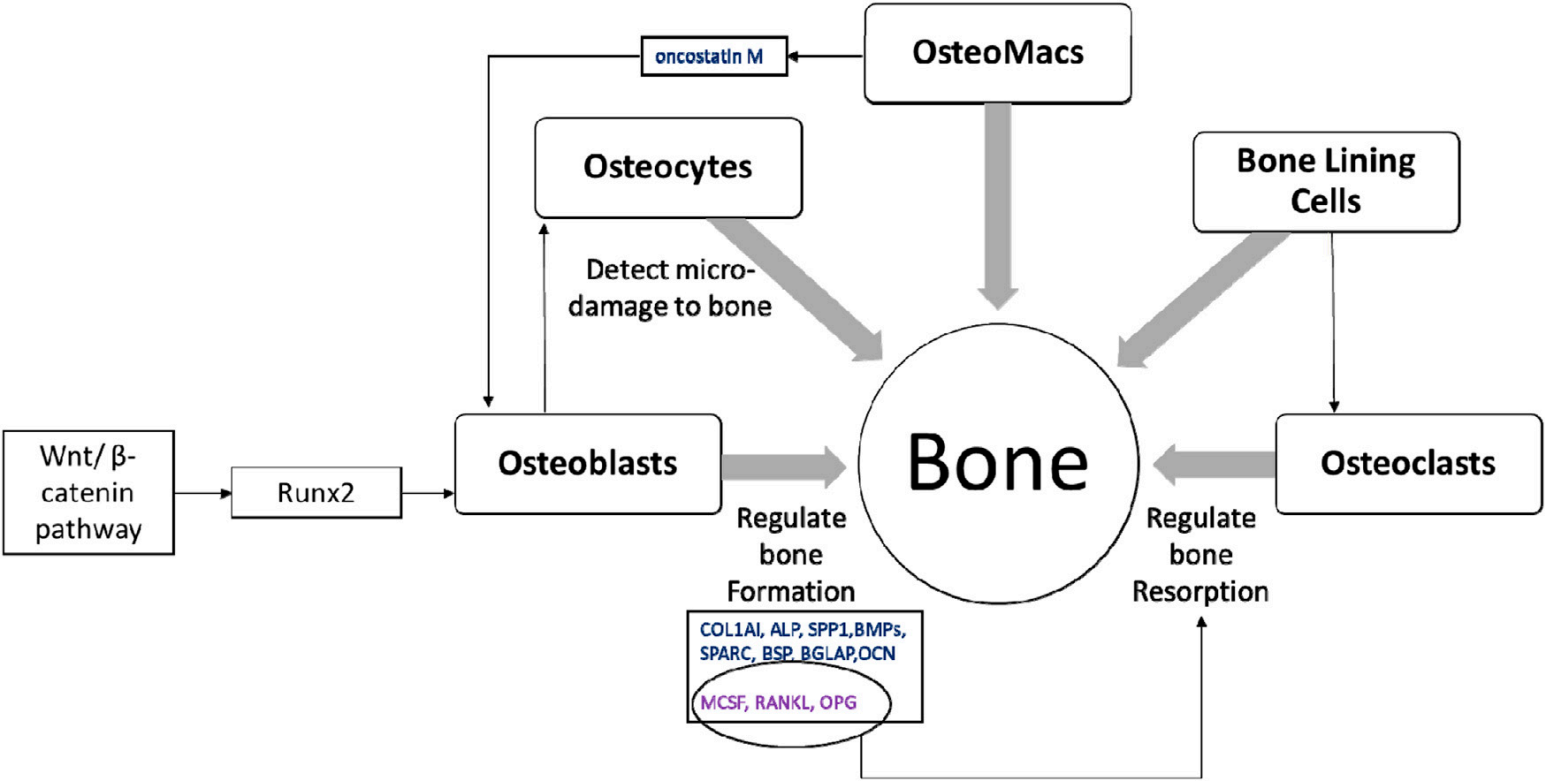
The multicellular nature of bone. Healthy bone is a result of balanced activity between osteoclasts and osteoblasts, which is regulated by different signalling pathways, transcription factors and cytokines along with other bone cells; osteocytes, osteomacs and bone lining cells (Takahashi et al., 1999; Guihard et al., 2012; Kular et al., 2012; Fakhry et al., 2013; Florencio-Silva et al., 2015; Gu et al., 2017; Kenkre and Bassett, 2018).

Bone is a multicellular organ that consists of different cell types such as osteoclasts, osteoblasts, osteocytes, bone marrow-derived macrophages or osteomacs and bone lining cells (BLCs) (Florencio-Silva et al., 2015; Miron et al., 2016). Osteoclasts are large, multinucleated bone-resorbing cells and are derived from hematopoietic stem cells. These cells secrete H+ ions, cathepsin K, tartrate-resistant acid phosphatase and matrix metalloproteinases directly onto the bone surface to effectively erode the mineral matrix. Osteoblasts are derived from mesenchymal stem cells or BLCs and chondrocytes; and are involved in bone formation (Rachner et al., 2011; Kular et al., 2012; Yang et al., 2014; Matic et al., 2016; Kenkre and Bassett, 2018). The activation of runt-related transcription factor 2 (Runx2) via Wnt/β-catenin pathway is important in osteoblast function resulting in bone formation (Brun et al., 2013; Langenbach and Handschel, 2013). Activation of Runx2 upregulates osteoblast-related genes such as collagen type I (COL1AI), alkaline phosphatase (ALP), osteopontin (SPP1), bone morphogenetic proteins (BMPs), osteonectin (SPARC), bone sialoprotein (BSP), bone gamma-carboxyglutamate protein (BGLAP), and osteocalcin (OCN) (Fakhry et al., 2013). Osteoblasts also produce macrophage colony-stimulating factor (MCSF), receptor activator of nuclear factor kappa-Β ligand (RANKL) and osteoprotegerin (OPG) to regulate osteoclast differentiation (Takahashi et al., 1999; Kular et al., 2012; Kenkre and Bassett, 2018). Osteocytes are mature osteoblasts that act as mechanosensors to identify bone microfractures while stimulating osteoclastogenesis, recruiting further immature cells from the surrounding pool to develop into a mature lineage (Gu et al., 2017). Further, osteocytes express sclerostin which is a negative regulator of bone formation (Wang J. S. et al., 2021). Osteomacs are bone resident macrophages involved in stimulating osteoblastogensis and matrix mineralisation by producing oncostatin M (Guihard et al., 2012; Gu et al., 2017). Inactive osteoblasts, (bone lining cells) are flat shaped cells that cover the area where no bone resorption or formation occurs. The function of bone lining cells is unclear, although they possess the potential to become active osteoblasts and regulate RANKL and OPG production to further regulate osteoclastogenesis (Florencio-Silva et al., 2015) (Figure 3).

### 2.3 Bone modelling and remodelling

**FIGURE 4:**
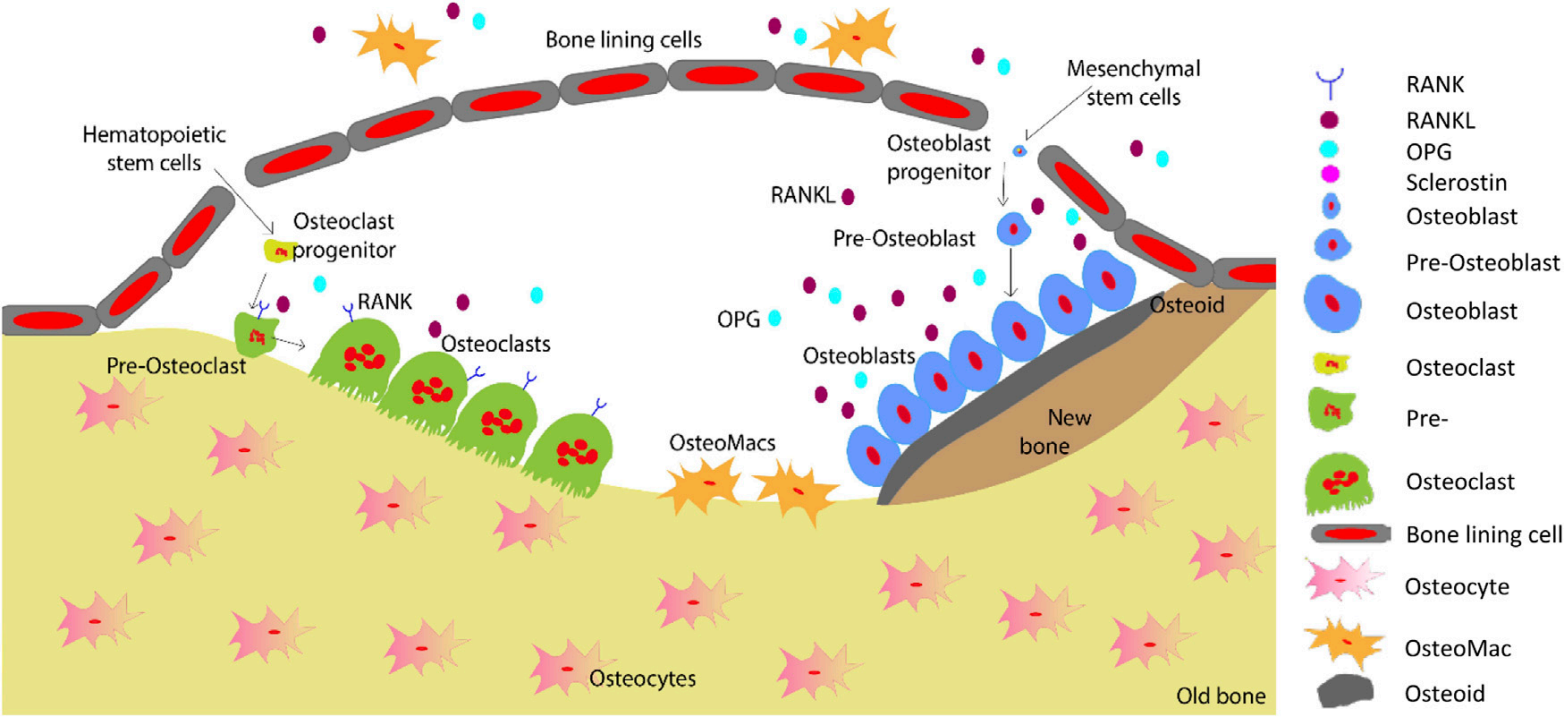
Schematic representation of the bone remodelling process. Osteocytes detect changes within their microenvironment and activate the bone remodelling cycle. Bone lining cells create a raised canopy above the remodelling surface. Osteoclasts migrate to the damaged area and resorb the bone followed by bone formation by osteoblasts. Osteomacs remove the remaining debris in the resorption compartment. The osteoblasts that are trapped in the matrix differentiate into osteocytes. Finally, remaining osteoblasts either go through apoptosis or turn into bone lining cells as the new bone forms (Saunders and Truesdell, 2019). Adopted from Saunders and (Saunders and Truesdell, 2019).

Bone regeneration is a complex skeletal maintenance and repair process via continuous bone formation (or modelling) and remodelling (Dimitriou et al., 2011). Bone modelling is the process that creates major volumetric and morphological changes to the bone structure, where bone resorption and formation occurs at an individual and distinct site (Seeman, 2009). Old and damaged bone is resorbed by osteoclasts, which is then replaced by osteoblasts (Figure 4). Imbalance in this bone remodelling cycle, whereby osteoclast resorption outweighs osteoblast bone formation, leads to bone defects such as decreased bone mineral density and increased fracture risk (Kenkre and Bassett, 2018). The bone remodelling process maintains the overall bone structure, with approximately one million active remodelling sites in the skeleton responsible for replacing approximately 10% of an adult’s bone each year (Manolagas, 2000; Kenkre and Bassett, 2018).

Bone remodelling occurs in basic multicellular unit (Kenkre and Bassett, 2018). Initially, micro damage to the bone is detected by osteocytes, which then recruit osteoclast precursors to the site of damage for resorption of that area to commence (Kenkre and Bassett, 2018). Osteoclasts resorb the collagen-rich matrix by secreting H+ ions and proteases such as cathepsin K and matrix metalloproteinase (Fuller et al., 2008). Next, osteoblasts initiate bone formation by secreting osteoid matrix, which is then mineralised (Kenkre and Bassett, 2018). Cytokines including RANKL, MCSF, OPG, interferons, interleukins, bone morphogenetic proteins and oncostain M are involved in the tightly controlled regulation of osteoclastogenesis and osteoblastogenesis (Fujiwara and Ozono, 2014; Amarasekara et al., 2021).

## 3 Causes of bone defects and typical treatments

The bone mass can be affected by a variety of conditions, including osteogenesis imperfecta, hyperprolactinaemia, hyperparathyroidism, rheumatoid arthritis, liver disease, myeloma, osteosarcoma, as well as the use of medications such as glucocorticoids, immunosuppressant, chemotherapy, anticoagulants, and antipsychotics (Oderda et al., 2012; Kenkre and Bassett, 2018; Azimi Manavi et al., 2023; Weerasinghe et al., 2023). Diseases such as osteoarthritis and osteosarcoma are leading causes of disability and have a major effect on the socioeconomic burden globally. Therefore, successful treatment is critical to reduce the burden of such diseases. A critical bone defect can be caused due to disease, trauma, surgery, developmental deformities and tumour resection leading to fractures where bone grafting is the preliminary treatment method (Wang and Yeung, 2017).

Bone defect treatment primarily focuses on improving bone regeneration, whereas treatment options vary based on the type and cause of the defect. Medications including anabolic, antiresorptive and antiosteoporotic treatment and hormone therapy are options to promote bone formation and bone mineral density (Langdahl et al., 2016; Shaheen et al., 2019; Gosset et al., 2021). Bone fractures typically include treatments such as stabilisation through casting or surgical intervention with bone grafting (Fillingham and Jacobs, 2016).

Bone grafting is a standard treatment option where it is the second most frequent tissue transplantation in clinical settings (Wang and Yeung, 2017). Bone grafts could be either autograft or allograft. Even though the success of these transplantation methods is higher than other options, major concerns are associated with bone grafting (Table 3). Due to the risks associated with bone grafting, including donor site mobidity, tissue mismatch and infection, bone graft substitutes have been developed. Commonly used approaches include, cell-based, ceramic-based, and polymer-based materials. Bone grafts substitutes aim to support osteogenic cells with osteo-inductive structures, vascularisation and mechanical properties (Henkel et al., 2013). However, none of the bone grafts substitutes can achieve all the desired properties for an ideal biomaterial.

**TABLE 3:** Current treatment and bone replacement options for bone defects and their advantages and disadvantages.

Treatment options for bone defects	Type of bone defect	Advantages	Disadvantages
Bone grafts			
Autologous	Facets of orthopaedic surgery, nonunions, posterior cervical fusions Pape et al. (2010); Sakkas et al. (2017); Pereira et al. (2020)	High success rate, osteoinductive, osteoconductive, osteogenic, no immune response after implantation, incorporate into its new site Pape et al. (2010); Sakkas et al. (2017); Pereira et al. (2020)	Limited availability, donor site morbidity, unpredictable resorption, vascularisation, pain in donor site, low immediate strength, low vascularity, risk of infectious disease transmission Pape et al. (2010); Sakkas et al. (2017); Pereira et al. (2020)
Allograft	Arthroplasty, spine, foot, hand, knee and trauma bone defects, tumour resection, prosthesis Bostrom and Seigerman (2005); Delloye et al. (2007)	No donor site morbidity, high availability osteoconductive osteoinductive, provide a structural framework/scaffold for host tissue to grow Delloye et al. (2007); Pereira et al. (2020)	Delayed incorporation, vascularisation, low availability of healthy grafts, rejection of the graft, risk of disease transmission, re-injury, ethical concerns Delloye et al. (2007); Pereira et al. (2020)
Xenograft	Dental implants Mello et al. (2020)	More economic, no donor site morbidity or pain, high availability, osteoconductive, osteoinductive, slow resorption rates, ability to define and maintain the volume for bone gain Mello et al. (2020); Pereira et al. (2020)	Limited osteogenicity, delayed incorporation, vascularisation, availability of healthy grafts, rejection of the graft, risk of zoonotic disease transmission, re-injury, ethical concerns Mello et al. (2020); Pereira et al. (2020)
Bone grafts substitutes			
Metals	Tibial defect, femoral defect, skull defect, mandibular defect, femoral condylar defect, ulnar defect, orbital defect Zhang et al. (2021)	Excellent mechanical properties, biocompatible, osteo-integration, personalised manufacturing, biocompatible, heat transduction Shayesteh Moghaddam et al. (2016); Pereira et al. (2020)	Stress shielding, corroding risk, risk of toxicity and initial inflammatory response, inadequate vascularisation Shayesteh Moghaddam et al. (2016); Pereira et al. (2020)
Ceramics	Primary and revision hip arthroplasty Whitehouse and Blom (2009)	Biocompatible, personalised manufacturing, good mechanical properties, resistance to Corrosion Pereira et al. (2020)	Brittle, low elasticity, inadequate vascularisation Pereira et al. (2020)
Polymers	Jawbone regeneration, ulnar bone defect Aoki and Saito (2020); Fraile-Martínez et al. (2021)	Biocompatible, personalised manufacturing, good mechanical properties, low young modulus Shayesteh Moghaddam et al. (2016); Pereira et al. (2020)	Lack of vascularisation, degrade by hydrolysis or erosion Shayesteh Moghaddam et al. (2016); Pereira et al. (2020)
Bone tissue engineering			
Hydrogels	Bone tissue repair, spinal cord injury repair, osteoarthritis cartilage damage repair, medical dressing, drug delivery Bai et al. (2018); Biopharma PEG Worldwide PEG Supplier (2022)	Provide nutrient environment for endogenous cell growth, mimic natural ECM, good mechanical strength Bai et al. (2018)	Mechanical weakness, limited control over degradation, lack of vascularisation Bai et al. (2018)

## 4 Alternative approach: Tissue engineering

**FIGURE 5:**
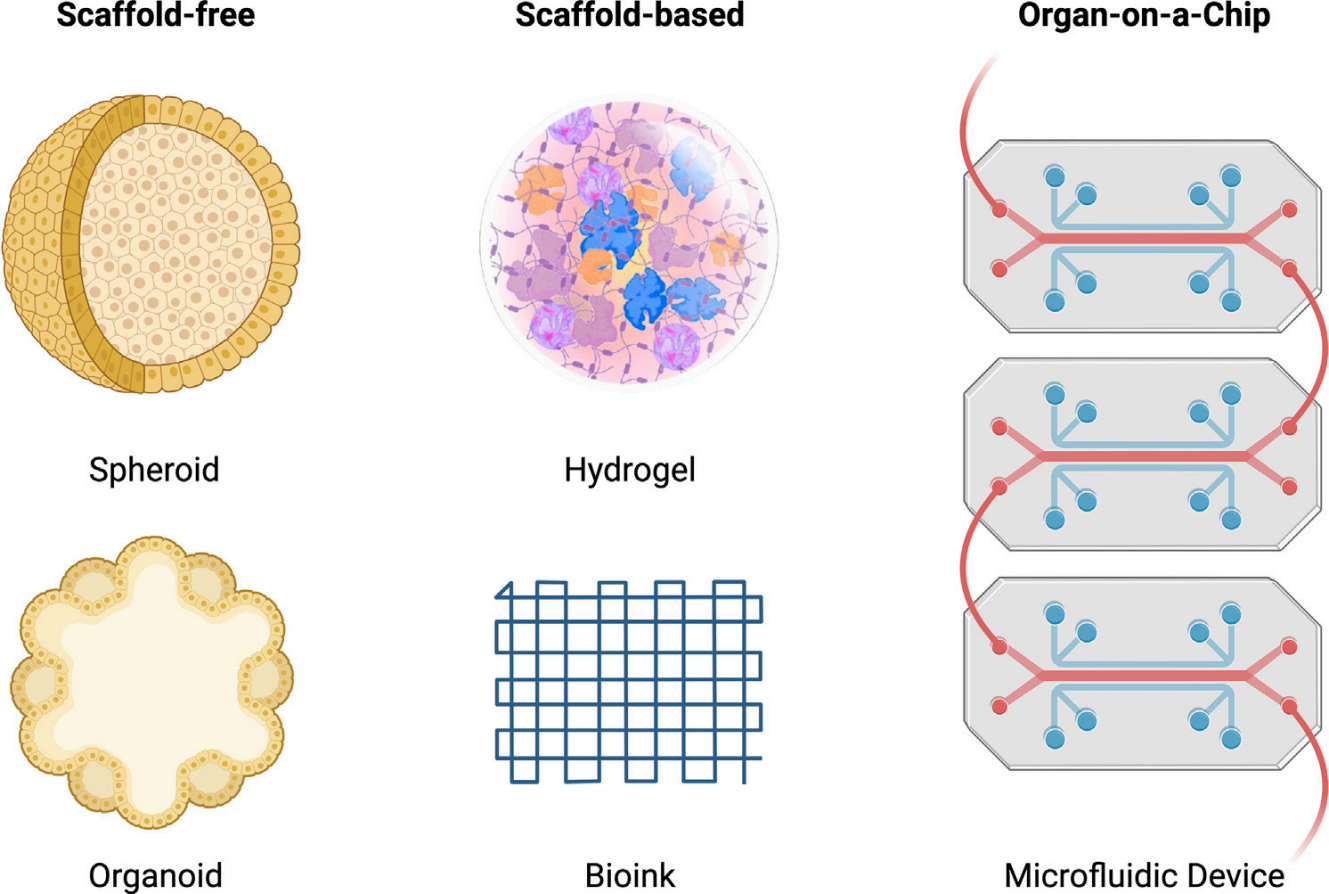
Schematic of scaffold-free, scaffold-based and organ-on-a-chip systems for tissue engineering. The scaffold-free systems include a spheroid and organoid model. The scaffold-based system includes a hydrogel and bioink model. The organ-on-a-chip system includes a microfluidic model.

Tissue engineering is a highly interdisciplinary field, aiming to restore, maintain and improve damaged tissues. The overall aim is to replace body parts with biomimetic replacements and ultimately recover full function. Tissue engineering combines three-dimensional constructs or scaffolds with biologically active molecules to induce bone repair, and regeneration provides an alternative solution for novel therapeutic outcomes with more accurate and reliable modelling of diseases to derive patient-specific outcomes. Figure 5 highlights the scaffold-free, scaffold-based and organ-on-a-chip systems discussed throughout this review.

The TE described here utilises the engineered biomaterials, cells, cues such as GFs, nanoparticles, or drugs, and fabrication modality to create a scaffold. To achieve biomimicry, the engineered tissue constructs need to have a complex hierarchical structure and resemble the ECM. Typical bone ECM contains polymeric matrix, composed of collagen type I and a mineral component composed mainly of hydroxyapatite (HAp). While unique to individual tissues, the ECM may be fine-tuned with building blocks including macromolecules and proteins such as collagen fibres, elastin, and proteoglycans organised in a tissue specific fashion (Theocharis et al., 2016). Natural tissues and organs exhibit both chemical and physical gradients in the architecture of the ECM, enabling many spatially diverse functional needs in a 3D matrix.

### 4.1 Biomaterial selection

The fabrication of a biomimetic material is an integral part of creating functional bone tissue. However, effectiveness of selecting an ideal biomaterial depends on its ability to interact with host cells and surrounding ECM. Biomaterials for bone regeneration can be characterised by the ability to promote osteogenesis and control the biological response of bone forming cells. A wide array of biomaterials such as metals, ceramics, polymers and hydrogels have been investigated for their potential to improve osseointegration and bone repair (LeGeros, 2008; Rodriguez et al., 2016; Heo et al., 2019; Amarasekara et al., 2021; Rifai et al., 2021). Hydrogels are three-dimensional scaffolds used in bone TE. Hydrogels can have a specific porous, highly hydrated structure, similar the native ECM, with good biocompatibility and biodegradability (Liu et al., 2022). Natural polymers such as gelatine, albumin, collagen, elastin, hyaluronic acid, chitosan, alginate and dextran can make hydrogels (Liu et al., 2022). Hydrogels are categorised as first generation, second generation, third generation and smart hydrogels (Yue et al., 2020). First-generation hydrogels are simple with a single polymer network while second-generation hydrogels can respond to environmental changes (Yue et al., 2020). Third generation hydrogels have enhanced and finely tuned physical properties while smart hydrogels can change their properties, for example, mechanical stability (Yue et al., 2020). However, hydrogels have challenges such as possible unintended immune responses, poor cell attachment, and degradation (Bai et al., 2018). Hence, there is need for improved TE approaches.

Hard biomaterials like metals have shown promise due to their excellent mechanical properties and they have been used for orthopaedic implants. However, lack of interaction with the bio-environment and inability of the inert metal to elicit osteogenic response has been a major challenge (Fong et al., 2021; Montoya et al., 2021; Wei et al., 2022). A recent study by Rifai et al. (2019) shows improved cell viability and proliferation as a result of the material’s surface properties. Thus, nanodiamond-coated titanium material compared to titanium alone shows better osteointegration potential after 3 days of cell growth due to the micro-rough surface and inherent carbon composition. (Mani et al., 2020; Fong et al., 2021). In another study, Heo et al. (2019) use collagen/fibrin hydrogels as an encapsulation matrix to embed human mesenchymal stem cells/human umbilical vein endothelial cell (MSC/HUVEC) spheroids to assess cell viability, morphology, proliferation and gene expression in comparison to cell suspension or MSC spheroid-laden hydrogels. The results show increased cell spreading and proliferation, upregulated osteogenic differentiation and pre-vascularised network in MSC/HUVEC spheroids embedded in collagen/fibrin hydrogels. Hence, a biomaterial mimicking the native ECM of bone provides conditions to assist the progression of the cell maturation pathway. To create interconnected structures facilitating the transport of GFs, it will be necessary to find a material that is stiff enough to support the mechanical needs of hard tissue-forming cells and porous enough to mimic 3D structures that resemble bone tissue. Additionally, a biomaterial that can be tuned will allow the material to respond to the physiological needs of the host tissue. Recent advances in biomaterials like hydrogels have created a 3D microenvironment that can closely simulate the ECM.

### 4.2 Cell selection

In addition to the type of biomaterial used for the success of tissue-engineered bone regeneration, the type of cells used in the biofabrication process are also important. Bone regeneration involves multiple cell lineages and requires cell proliferation, differentiation and maturation. Studies indicate that bone marrow derived MSCs, adipose tissue derived MSCs and periosteal stem cells are involved in bone healing processes with higher osteogenic potential (Ono and Kronenberg, 2016; Shen et al., 2019). Bone marrow derived osteoprogenitors can be differentiated into osteogenic lineage *in-vitro* and have pivotal role in bone repair (Friedenstein et al., 1987). Similarly, Moncal *et al.*, (Moncal et al., 2022), show rat bone marrow mesenchymal stem cells can be loaded in a bioink and intraoperatively delivered into a craniomaxillofacial defect site. The bio-printed bone constructs show ∼40% bone tissue formation and ∼90% bone coverage area at 6 weeks compared to ∼10% new bone tissue and ∼25% total bone coverage area in empty defects. Adipose tissue derived MSCs possesses the ability to differentiate into variety of cells with immunomodulatory, osteoinductive and anti-inflammatory features making it as a good source for bone repair and regeneration (Zhang et al., 2022). Adipose tissue derived MSCs have currently tested in bone transplantation with combination of scaffolds to improve bone induction and remodelling in rodents (Xu et al., 2019; Wang Z. et al., 2021). A study conducted by Conejero *et al.*, showed that adipose derived MSCs can be used to repair rat palatal bone defects (Conejero et al., 2006) while Shin *et al.*, showed the use of adipose derived MSCs in rotator cuff repair; a common sports injury (Shin et al., 2020). The periosteum is a thin, vascular tissue covering the surface of the bone and *in-vitro* studies confirm the periosteum to contain MSCs/skeletal progenitor cells (Nakahara et al., 1991). Lineage tracking experiments during bone healing denote the periosteum as a primary source of bone forming cells in the fracture callus (Murao et al., 2013; Roberts et al., 2015; Bahney et al., 2019). A study reports periosteum-derived mesenchymal progenitors have better bone forming capacity than bone marrow-derived progenitors *in-vivo* (Radtke et al., 2013). Even though we do not yet have a specific cell marker to identify and investigate periosteal progenitor cells for clinical use, periosteal stem cells remains among the most promising cell types for TE studies (Roberts et al., 2011).

### 4.3 Selecting growth factors

Growth factors and cytokines are typically used within scaffolds to create a dynamic system to guide cell development. Osteoblast activity is regulated in an autocrine (a form of signal where cells secrete a hormone or a chemical that binds to the receptors on the same cell) and paracrine (where the signal is released to a nearby target cell) manner. The activity is controlled by GFs including IGF, PDGF, FGF, TGFβ, and BMP, whose receptors have been found on osteoblasts. On the other hand, osteoclasts resorb bone by acidification and proteolysis of the bone matrix and hydroxyapatite (Hadjidakis and Androulakis, 2006). Osteoclast function is regulated by systemic hormones and locally acting cytokines. For bone remodelling, both osteoblasts and osteoclasts work together. Due to the complexity of GFs involved in the natural process of bone formation, resorption and remodelling, the TE system has to be more dynamic to allow cells to progress along the lifecycle of bone. Typical soft-tissue materials tend to focus on delivering BMP and VEGF to induce bone growth and vascularisation, respectively. (Orciani et al., 2017). The ideal biomaterial mimics the ECM by serving as a storage site for GFs and regulate growth factor production and signalling (Wilgus, 2012).

Different TE approaches have been employed to functionalise biomaterial scaffolds with bioactive compounds including physical encapsulation, bioaffinity and covalent immobilisation of GFs to the material scaffold (Wang et al., 2017). Various GFs, as detailed in Table 1, are responsible for the regulation of bone regeneration due to their potent effect on bone cell metabolism (Devescovi et al., 2008b). For instance, Moncal *et al.,* investigate the possibility of deriving an osteogenic bioink from the co-delivery of plasmid DNAs encoded with BMP-2 and platelet-derived growth factor-B. The growth factor encoded plasmid-DNAs form printed bioinks within 6 weeks and provide a significantly higher formation of bone tissue and area coverage compared to the control bioinks. (Moncal et al., 2022; Mahmoudi et al., 2023). Moreover, Freeman et al. (2020) show that the level of angiogenesis *in vivo* is reliant on the spatial presentation of VEGF. Large bone defects show accelerated healing with printed implants that contain a VEGF gradient and BMP2 localisation. In comparison to physical entrapment, chemical immobilisation can overcome the initial burst release. (Nyberg et al., 2016; De Witte et al., 2020). More importantly, the process often involves chemical/enzymatic reaction between GFs and the scaffold, which in turn provides opportunity to develop controlled, localised, and sustained release systems. In another study, Park et al. (2020) developed a click-crosslinked hydrogel scaffold with BMP2 mimetic peptide (BP). They show that the chemically loaded hydrogel scaffold retained the BP for over 1 month. The BP successfully induced osteogenic differentiation in human dental pulp stem cells. The use of chemical coupling approaches enables GFs to covalently bind to the scaffold, as such, Cho et al. (2014) show polydopamine assisted tethering of BMP2 and Li *et al.* (Li et al., 2014) show streptavidin assisted binding of BMP2 to methacrylic chitosan polymer. Other studies suggest that utilising GF binding motifs of adhesive protein in the ECM including fibronectin, laminin, fibrinogen, and collagen are important for mimicking the ECM. (Dhavalikar et al., 2020). Furthermore, the hierarchical structure and architecture of the ECM microenvironment can be exploited for effective GF release system Hence, growth factors serve to functionalise scaffolds, better facilitating the requirements of the native tissue.

### 4.4 Creating a 3D-microenvironment

Due to the quad of motifs required to create a biomaterial, attempts have typically sought to initiate a cycle of enhancing the cell’s innate ability to form tissues, thus creating a ‘scaffold-free’ system *in-vitro* that enables researchers to map the capability of precursor tissues to restore targeted native development *in-vivo*. It is now possible to leverage the cell’s own capacity to synthesise an ECM with the appropriate exogenous cues including biophysical and chemical factors. The principle of self-organisation refers to the result of external forces in a system (Athanasiou et al., 2013). In comparison, self-assembly refers to the spontaneous arrangement of cells through cell-to-cell interactions. Typically, self-assembly does not require an adherent substrate. In contrast, self-organisation can employ methods such as TE to engage cells to aggregate and be positioned during important events of embryonic development.

**FIGURE 6:**
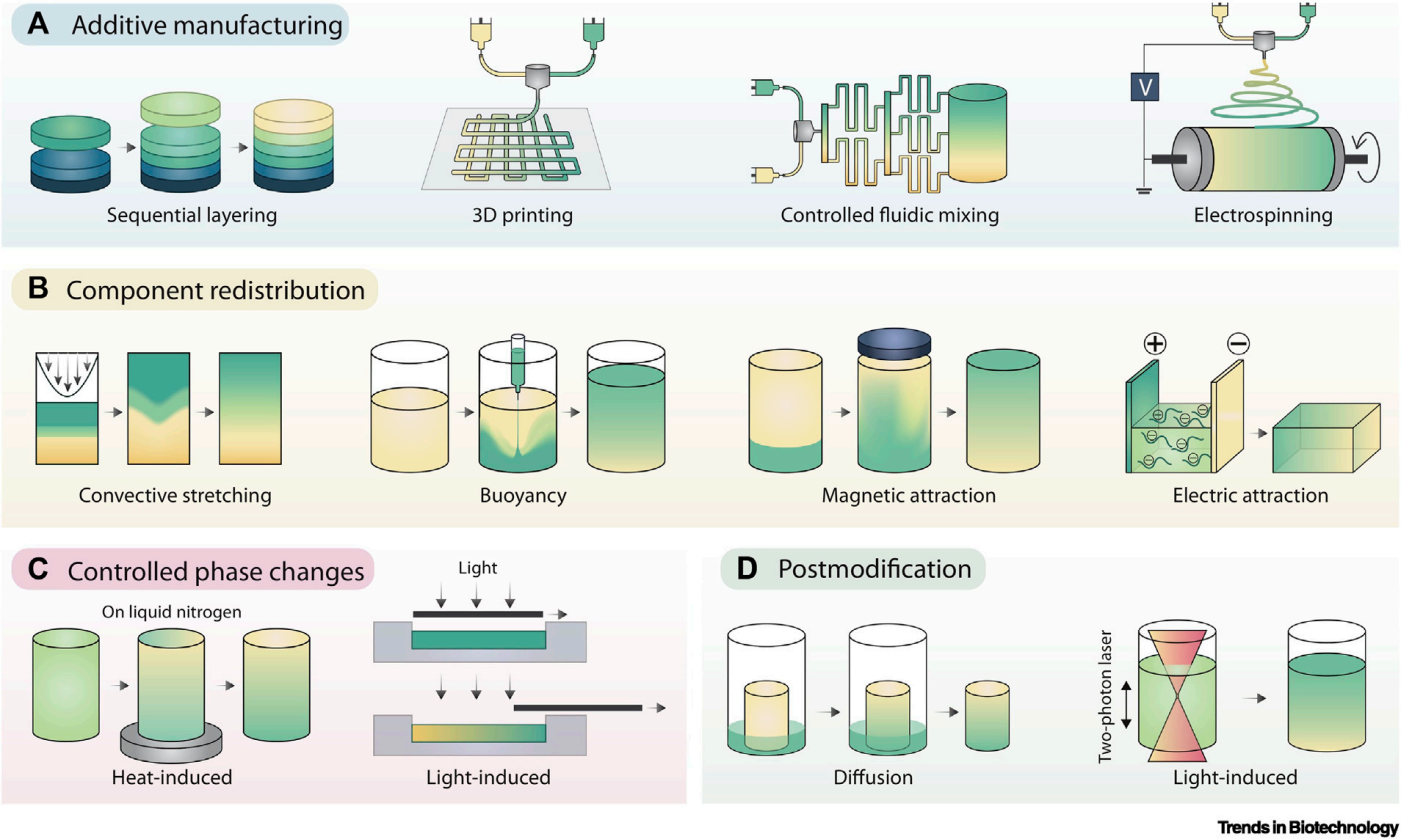
Gradient Fabrication. **(A)** Additive manufacturing is an intuitive approach to gradient fabrication, with methods including sequential layering, 3D printing, controlled fluidic mixing, and electrospinning. **(B)** Component redistribution approaches produce gradients from an initially homogenous distribution by controlled demixing via convective stretching, buoyancy, magnetic fields, or electric fields. **(C)** Controlled phase changes can also result in forming gradients from homogeneous starting materials, typically using graded exposure to heat or light. **(D)** Post modification involves the presentation of a gradient onto preformed materials, typically achieved by controlled component diffusion or photopatterning. Adopted with permission from Li et al. (2021).

When cells have a truly 3D environment, they will enter a stage of cell fusion. A number of methods including gradients-additive manufacturing can be employed, including microfluidics and electrospinning (Figure 6). These techniques will enable different types of scaffold architecture to be developed within one unit. Multiple cells can form microtissues to resemble a state of native development *in-vivo*. As such, engineered organoids are unrivalled with regards to their potential to form tissue analogues to those found in organs. However, the key issue is the ability to scale-up and reproduce large numbers to create regenerative outcomes.

### 4.5 Fabrication of functional bone tissue

To overcome challenges in fabricating tissues at defect sites, biofabrication is used as a method of rapid and intricate manufacture. Combining the relevant cellular cues and scaffold will enable a wide range of outcomes including a functional musculoskeletal system. The musculoskeletal system has been well studied to date with a number of different cell types. By leveraging 3D printing strategies, it is possible to modulate the architecture of engineered bone-tissue grafts to enhance their capacity to regenerate large bone defects. Furthermore, several biofabrication tools can be used to engineer a mechanically reinforced bone-tissue template without negatively impacting its capacity to support the development of a vascularised bone organ *in-vivo*. Despite the potential of biofabrication, a number of parameters must be addressed before the technology can fully be realised.

### 4.6 Bioprinting in supporting bath: FRESH

**FIGURE 7:**
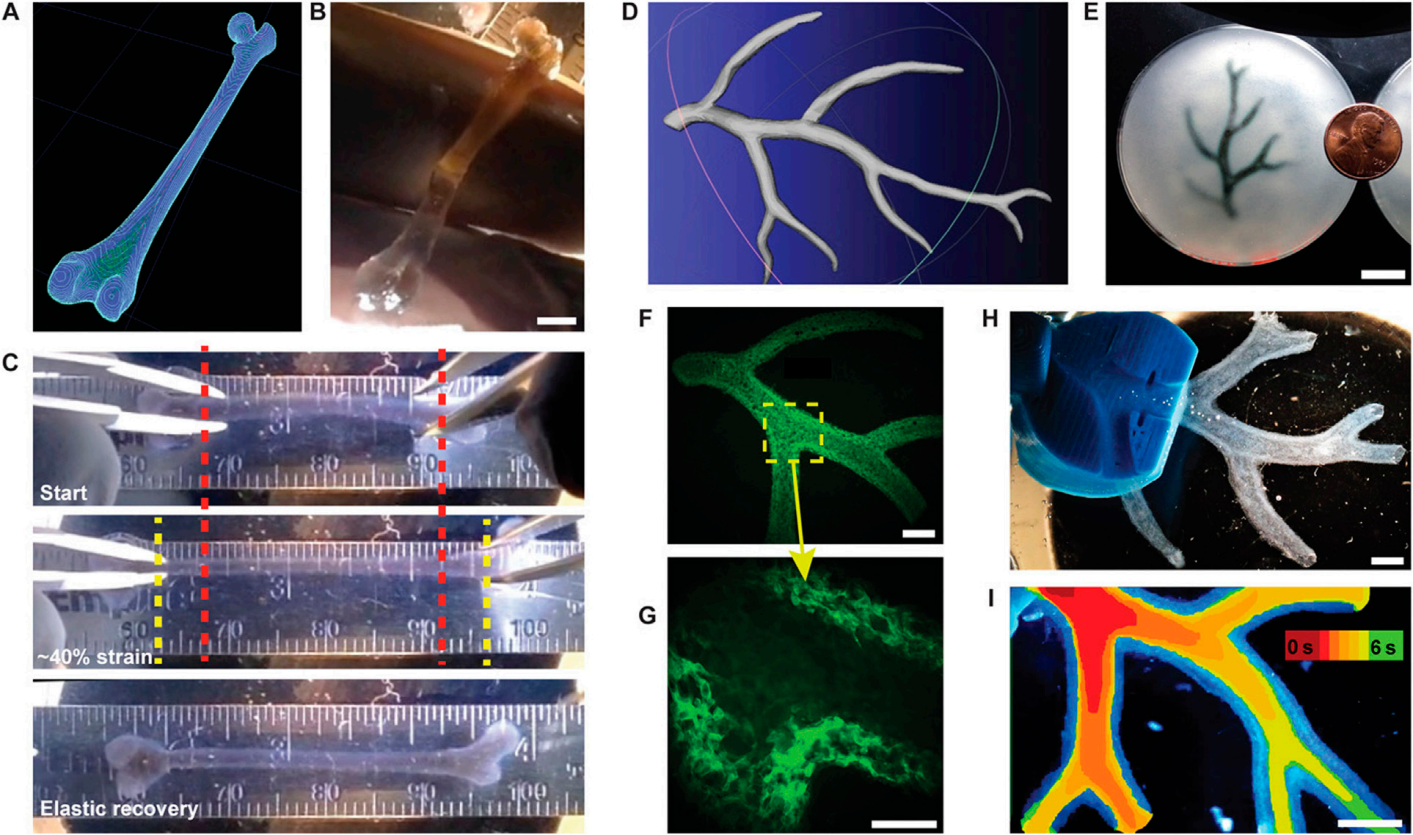
FRESH printing of biological structures based on 3D imaging data and functional analysis of the printed parts. **(A)** A model of a human femur from 3D CT imaging data is scaled down and processed into machine code for FRESH printing. **(B)** The femur is FRESH printed in alginate, and after removal from the support bath, it closely resembles the model and is easily handled. **(C)** Uniaxial tensile testing of the printed femur demonstrates the ability to be strained up to 40% and elastically recover. **(D)** A model of a section of a human right coronary arterial tree from 3D MRI is processed at full scale into machine code for FRESH printing. **(E)** An example of the arterial tree printed in alginate (black) and embedded in the gelatin slurry support bath. **(F)** A section of the arterial trees printed in fluorescent alginate (green) and imaged in 3D to show the hollow lumen and multiple bifurcations. **(G)** A zoomed-in view of the arterial tree shows the defined vessel wall that is < 1 mm thick and the well-formed lumen. **(H)** A dark-field image of the arterial tree mounted in a perfusion fixture to position a syringe in the root of the tree. **(I)** A time-lapse image of black dye perfused through the arterial tree false-coloured at time points of 0–6 s to show flow through the lumen and not through the vessel wall. Scale bars, 4 mm **(B)**, 10 mm **(E)**, 2.5 mm **(F)**, 1 mm **(G)**, and 2.5 mm **(H, I)**. Adopted with permission from Hinton et al. (2015).

Recent work by Hinton et al. (2015) show the capabilities of printing soft materials including hydrated alginate, collagen, and fibrin, taking hydrogel research to new platforms (Figure 7). The technique is termed freeform reversible embedding of suspended hydrogels (FRESH). The study leveraged the knowledge and capabilities of freeform printing. The FRESH technique uses a slurry of microparticle baths as a secondary hydrogel and high shear thinning nozzle to maintain the 3D-geometry and print an embedded hydrogel. The process takes place in sterile aqueous conditions, at 37°C in buffered environments, meaning that cells can maintain their viability and are compatible with the extruded environment. Hoch et al. (2016) report that the phenotype of cells can be maintained by including the ECM with cell-secreted enzymes. They show that depositing osteogenically-induced MSCs on cell-secreted, decellularised ECM produces up to 2-fold more calcium deposition than tissue culture plastic. This enhancement is partly related to increased actin cytoskeletal tension via the ROCK II signalling pathway. This indicates that even after removing induction stimuli, such as differentiation, the physiological milieu can affect cell bioactivity and fate for prolonged periods of time.

In contrast to the FRESH method, Wu et al. (2011) describes 3D printing of a hydrogel ink within a hydrogel support bath for omnidirectional printing, which is an ink designed to leave micro channels within a permanent support bath. The support bath is then crosslinked with ultraviolet afterwards to repair the damage caused by the nozzle.

### 4.7 Aspiration-assisted bioprinting

**FIGURE 8:**
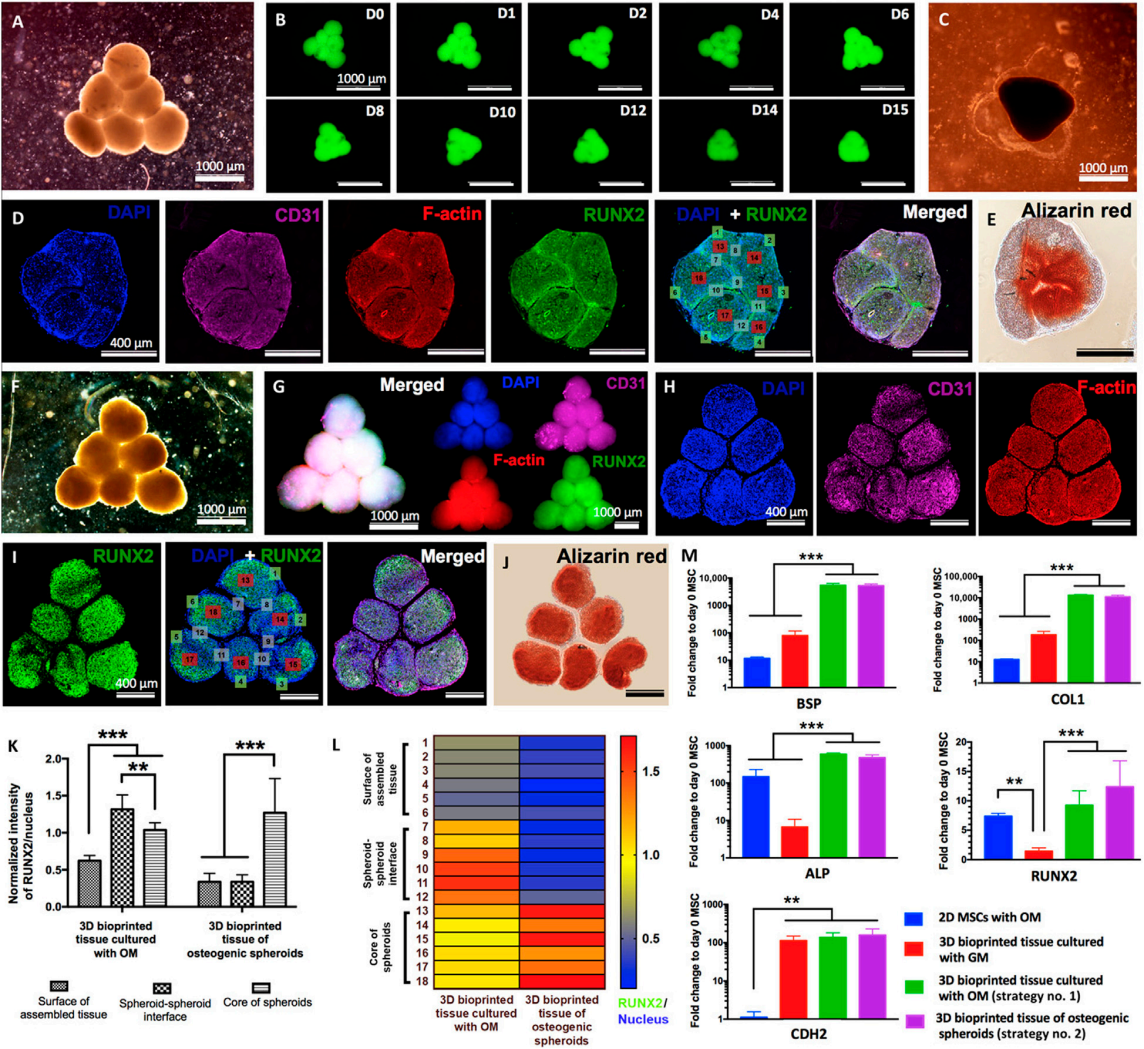
Biofabrication of osteogenic tissues. Strategy no. 1: **(A)** Triangle-shaped tissue complexes were bioprinted using MSC/HUVEC spheroids and cultured for 3 days in GM and 12 days in OM. **(B)** Time-lapse images showing fusion of GFP^+^ spheroids up to day 15 (D15) after bioprinting. **(C)** An optical image showing the assembled tissue at day 15 after bioprinting. **(D)** Immunofluorescence staining (DAPI, CD31, F-actin, RUNX2, and DAPI + RUNX2) and **(E)** Alizarin red staining of the sectioned tissue. Strategy no. 2: **(F)** The final shape of the bioprinted tissue of osteogenic spheroids (cultured for 10 days in OM before bioprinting and 2 days in OM after bioprinting). Immunofluorescent images of **(G)** the bioprinted tissue and **(H)** confocal images of its histological sections stained for DAPI, CD31, and F-actin and **(I)** RUNX2 and DAPI + RUNX2. **(J)** Alizarin red staining of the tissue section. **(K)** Quantification of normalized RUNX2 intensity at different regions including the surface of assembled tissue, spheroid-spheroid interface, and core of spheroids (*n* = 50; **p < 0.01 and ***p < 0.001). **(L)** A representative heat map figure showing RUNX2/DAPI distribution in the surface of assembled tissue, spheroid-spheroid interface, and core of spheroids for strategy nos. 1 and 2. **(M)** *BSP*, *COL1*, *ALP*, *RUNX2*, and *CDH2* gene expressions of 2D MSCs cultured in OM (control), 3D bioprinted tissues cultured in GM (control), and 3D bioprinted tissues cultured using strategy nos. 1 and 2 (*n* = 5; **p < 0.01 and ***p < 0.001). Adopted with permission from Ayan et al. (2020).

Aspiration-assisted bioprinting provides the opportunity to control the position of tissues in 3D-space. The technique uses the power of aspirational forces with microvalve bioprinting. Ayan et al. (2020) demonstrate that a wide range of biologics including tissue spheroids (80–600 μm), tissue strands (∼800 μm), or single cells (electrocytes, ∼400 μm) could be printed. They used patterns to form sprouting spheroids for angiogenesis and self-assembly to form osteogenic spheroids (Figure 8). In addition, microwell stamps by Freeman et al. (2022) show that tumour spheroids modelled with the drug Doxorubicin could be used to understand the relationship between tumour elimination and bone regeneration. Osteogenic supplements had minimal effect on cancer cell growth but stimulatory effect on stromal cells. However, the stimulatory effects of the osteogenic supplements were not observed when delivered with chemotherapeutics. A more natural biological setting is being emulated by 3D microenvironments, including self-assembly through cell-to-cell contact. The heterogenous nature of spheroids allow cells to develop a rigid 3D structure. The hydrogel provides a stable environment with exogenous cues for the cells to respond in a differentiated form with or without a scaffold configuration. In general, MSCs cultured in osteogenic media express high levels of osteogenic markers. To confirm the osteogenic differentiation of bioprinted tissues, Ayan et al. (2020) use *RUNX2*, the early osteogenic differentiation marker for staining. Calcium deposition of osteogenically differentiated tissues was also confirmed by Alizarin red staining. As shown in Figure 8, substantial calcium deposition was observed after 12 days of osteogenic induction with the Alizarin stain. The expression levels of *BSP*, *COL1*, *ALP*, and *RUNX2* genes were similar and significantly higher compared to control groups. Overall, by altering the osteogenic timeframe under the same total osteogenic induction duration, the shape of bioprinted tissues and mineralisation could be guided. However, no differences were detected in the expression levels of osteogenic genes. In another report, Westhrin et al. (2015) show that both ALP-modified and unmodified alginate beads provide an osteogenic condition compared to two-dimensional environments. The MSCs expressed higher levels of osteogenic markers including *RUNX2*, *COL1A1*, osterix (*SP7*) *BGALP* than the cells in traditional cell cultures.

### 4.8 Constructs and bioinks

**FIGURE 9:**
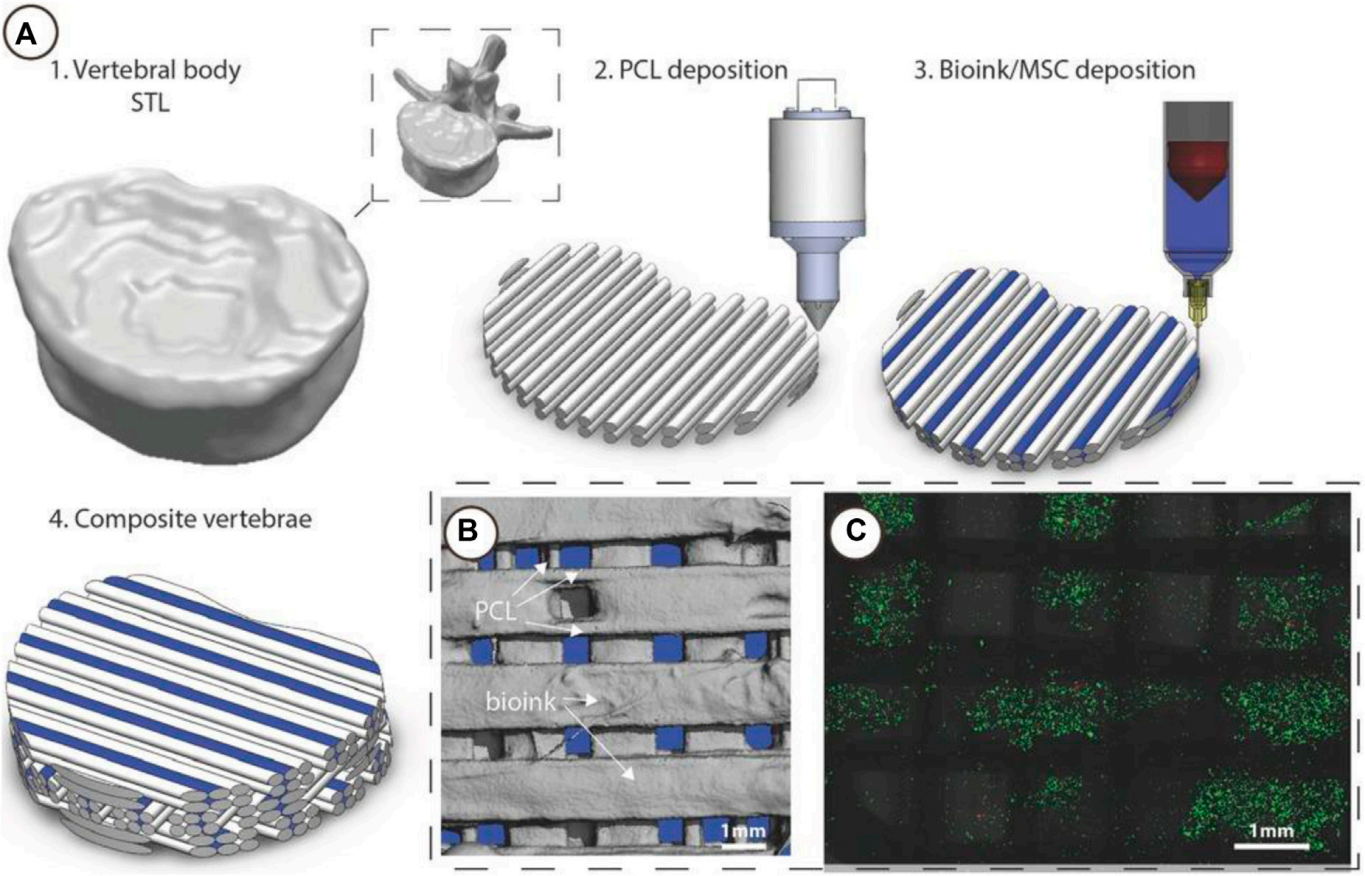
3D Bioprinting of vertebrae-shaped mechanically reinforced bioinks. **(A)** Description of multi‐tool 3D bioprinting process, 1) The outer geometry of a human vertebral body was scanned and next layers of 2) PCL filaments were deposited followed by deposition of the 3) MSC laden bioink, this was repeated in an orthogonal fashion to create a 4) composite vertebrae structure. **(B)** μCT analysis demonstrated the distribution of bioink and PCL within the composite vertebrae. Bioink + PCL filaments isolated using μCT, indicating the presence of bioink free channels conduits (blue regions) post‐printing. **(C)** Live/dead images of cells within the deposited bioink 1 h post‐printing, scale bar 1 mm. Adopted with permission from (Daly et al., 2016).

One of the major challenges at the musculoskeletal interface is to recreate a mechanically stable scaffold. Daly et al. (2016) show that a polycaprolactone (PCL) bioink scaffold improves cell viability (Figure 9). The bioinks show that the cells are functional. Likewise, Alcala-Orozco et al. (2022) show that they can print complex multi-material constructs including a bioink and thermoplastics. In their *in-vitro* study, they show Mg-PCL/Sr-GelMA hybrid constructs cultured in osteogenic media for 21 days. The expression of the osteocalcin and collagen I bone markers correlate to osteogenic differentiation. Further characterisation with Alizarin red staining shows calcium deposition. Therefore, the use of bioinks with mechanically robust materials such as PCL, provide structural support for high load bearing bone-tissue applications. The investigation of combining multiple materials may become useful when using FDA-approved materials for translational outcomes. However, several challenges in understanding cellular mechanisms still exist, particularly with complex biomaterials and the parameters involved.

### 4.9 Microfluidic bioprinting

Over the past few years, the widespread adoption of 3D printing has opened up numerous opportunities for utilising this technology in the development of 3D-printed organ-on-chip models. By integrating 3D printing with microfluidic technology in organ-on-chip systems, researchers have a more efficient approach for constructing intricate flow channels and chambers. Furthermore, this amalgamation enables the creation of biologically relevant structures with 3D cell distribution and biomaterials, preventing cell damage during printing, maintaining heterogeneity, and tissue-specific functionality. New methods of modifying printing nozzles with microfluidic designs are typically employed to contrast intricate systems.

Vascularisation plays a crucial role in supplying nutrients, oxygen, and removing waste from living tissues and organs. However, achieving vascularisation in engineered tissue and organ constructs remains a significant challenge. Zhang et al. (2013) utilise a microfluidic needle to 3D-bioprint vascularised tissue and organ constructs. These constructs consist of hollow fibres that mimic blood vessels, with cell constructs encapsulated within the fibre walls. This approach not only provides mechanical stability but also allows for fluid transport within a 3D cellular environment. (Nashimoto et al., 2017; Williams et al., 2022). They show that cartilage progenitor cells encapsulated in the hydrogel walls are viable during prolonged periods of *in vitro* culture and express cartilage-specific genes. A study by Lee et al. (2014) uses a layer-by-layer approach, printing collagen and gelatin fibres, and then melting the gelatin to expose channels. Bertassoni et al. (2014) use agarose as the sacrificial layer material, enabling the construction of fully perfusable microchannels within a cell-laden hydrogel bulk.

These studies demonstrate the diverse sacrificial layer materials and techniques employed to achieve the bioprinting of intricate structures and functional tissue constructs, offering potential applications in TE and drug screening. Additionally, the integration of microfluidic control with bioprinting techniques opens up possibilities for functionally graded additive manufacturing, involving the use of multiple biomaterials and/or multicellular structures. This approach holds great promise for future 4D bioprinting advancements.

## 5 Challenges and future outcomes

The current gold standard in clinical bone-tissue replacement is bone-grafts, with allo- and auto-grafts. In spite of this, it is not ideal due to the challenges mentioned previously. With the exciting prospect of TE, designing a biomaterial, encompassing osteoconductive, osteoinductive, and osteogenic properties will provide better treatment solutions to integrate into the body, and allow the host cells to regenerate.

An emerging field, biofabrication, integrates additive manufacturing-derived bioprinting methods with biological and engineering self-organisation techniques. Recent biofabrication techniques explored in this review demonstrate the limited ability to guide cell arrangements and tissue structures. They promise to generate complex and heterogeneous tissues with further developments. There is immense potential for applications in drug development, toxicology screening, cosmetics, and disease modelling platforms. Arbitrary large-scale structures (>1 mm) can be produced. However, researchers are still working on creating defined microenvironments that mimic native tissues. The convergence of different biofabrication strategies for hybrid approaches is currently relevant to identify approaches that are suitable for tissue and cell types at different length scales. Hence, the advances in machine learning and predictive models will most likely provide great insight into ‘*Digital twins’*, which refers to a digital model of a real-world system or in this case, additive manufacturing protocols. This digitisation could potentially help validate a plethora of drugs and material compatibility. Biological, chemical, and physical factors that drive tissue regeneration can be understood by investigating how cells respond to microenvironments with defined control over phenotypic responses. Although biofabrication is progressing rapidly, functionality of bioprinted tissues has not reached therapeutic scales and dimensions. Significant developmental work is required before bioprinted tissues find widespread use.

One of the major challenges associated with TE scaffolds is that they should test for their bioactivity and mechanical and chemical properties using both *in-vitro* and *in-vivo* models. The *in-vitro* models identify cell function, biological activity, toxicity and immunogenicity. However, *in-vitro* models are not capable of reflecting the complex *in-vivo* environment. The *in-vivo* rodent models also have some drawbacks such as relatively small size for testing implants, the lack of harversian canal systems, minimal intra cortical remodelling, smaller proportion of cancellous bone to total bone mass and different skeletal loading patterns to name some (Henkel et al., 2013).

Research in TE continues to advance towards modelling bone tissue’s complex structure (Zhou et al., 2019; Freeman et al., 2020; Rifai et al., 2021; Burdis et al., 2022). There are gaps in science pertaining the hierarchical and anisotropic nature of bone, particularly in terms of vascularisation and development physiology. Currently, biomaterials that are designed to be implanted, target the delivery of cytokines or GFs. It is important that the stages of bone healing and remodelling are understood thoroughly to have impact when it is required. Otherwise, there may be adverse effects, causing inflammatory responses that may interfere with the natural bone healing process. Regardless of the manufacturing procedure, vascularisation is typically ignored when designing complex biomaterials. However, developing scaffold with functional properties for bone defects to allow more complex and intricate parameters for vascularisation need to be considered. Likewise, appropriate models of bone healing and regulating factors, including immune cell response for bone regeneration need to be studied.

New TE approaches are investigating patient cells *ex-vivo* to stimulate tissue regeneration. This enables the design of the scaffold using the patient’s own cells, which can then be re-implanted. The implant scaffold should be able to support new tissue develop, while simultaneously disintegrating over the time, hence leaving no harmful stimulus at the implantation site. However, translation of these advanced TE strategies to clinical practices is challenging due to the available funding and gaining approvals from regulatory bodies for safety.

Furthermore, from a manufacturing perspective, TE has risen over the past decade to create biomimetic 3D constructs. Although scaffolds lack clinical relevance, a certain level of spatiotemporal control over cell development has been observed. To improve the direction of scaffold manufacture towards clinically relevant tissues, the following should be considered: 1) design principles including anisotropic and gradient scaffolds; 2) tunable biodegradability to match the cell growth, differentiation and maturation, 3) precise control over the delivery of cytokines and drugs; and 4) vascularised cellular networks. These developments have wide-ranging applications in TE, organ transplantation, regenerative therapy and high-throughput drug screening.

## 6 Conclusion

With the growing field of TE, a deeper understanding of the native bone tissue and the urge to regenerate specific interfaces are becoming more prevalent. This review highlights the technological advancements to create complex and intricate bone tissue-engineered structures. While advances have been made with synthetic approaches, there are still gaps in improving the functionality of tissue-engineered scaffolds, particularly in predicting cell growth, differentiation, and maturation. The field lacks clinical relevance and translation prospects, where only very few systems highlight the prospects of vascularisation. Recent studies focus on developing osteogenically differentiated scaffolds prior to gel encapsulation. The use of hydrogels enables the creation of miniaturised tissues similar to native tissues, including aggregates, spheroids, and organoids. It will be worth pursuing dynamic systems to regenerate anatomically and physiologically relevant tissues. Hydrogels with cues to respond to the human body’s innate microenvironment hold promise to govern the development of new biomaterials in tissue engineering.
